# Effect of vibration during bulk and incremental filling on adaptation of a bulk-fill composite resin

**DOI:** 10.1038/s41598-022-26197-9

**Published:** 2022-12-15

**Authors:** Yung-Soo Kim, Seung-Ho Baek, Ryan Jin Young Kim

**Affiliations:** 1 School of Dentistry, Seoul National University, 101 Daehak-ro, Jongno-gu, Seoul, Republic of Korea; 2 Department of Conservative Dentistry, Dental Research Institute, School of Dentistry, Seoul National University, 101 Daehak-ro, Jongno-gu, Seoul, Republic of Korea; 3 Dental Research Institute, School of Dentistry, Seoul National University, 101 Daehak-ro, Jongno-gu, Seoul, Republic of Korea

**Keywords:** Dental equipment, Bonded restorations, Composite resin

## Abstract

This study evaluated the effect of vibration on adaptation of bulk-fill composite resin. A scanning laser doppler vibrometer measured the frequency and amplitude of a vibratory device (COMO; B&L Biotech) used for resin placement and visualized its effect on the resin according to depth. A bulk-fill composite resin (Filtek Bulk Fill; 3M ESPE) was placed in simulated cavities (4 mm diameter, 4 mm depth) by different layering methods (incremental filling with two 2-mm-thick layers vs. bulk filling with a single 4-mm-thick layer). The groups were further divided based on the application of vibration during restoration (no vibration vs. vibration). In addition to the surface void area at the cavity floor, the overall void volume and the void volumes of the bottom, middle, and top thirds were obtained for micro-computed tomography analysis. The frequency and amplitude of the COMO were approximately 149 Hz and between 26 and 51 µm, respectively. When vibration was not applied, incremental filling had a lower void volume in the bottom third of the cavity than did bulk filling (*p* < 0.05). Vibration applied with a 4-mm-thick bulk fill had no significant effect on the adaptation of composite resin (*p* > 0.05). In contrast, vibration reduced the amount of void formation in the bottom third of the cavity during incremental filling (*p* < 0.05). Application of vibration to resin with a 2-mm incremental-layering technique formed a smaller void at the interface between the cavity and resin and within the bulk-fill composite resin.

## Introduction

Composite resins are the most widely used direct restorative dental materials because of their aesthetics and ability to adhere to teeth with proper bonding materials. However, composite resins inevitably shrink by 2–4% during polymerization^1^, which can cause stress-induced debonding at the tooth-restoration interface when shrinkage stress exceeds bond strength^2^. Incremental filling, in which each layer of a 2-mm-thick composite is light-cured, is recommended to minimize potentially detrimental polymerization shrinkage stress^3,4^. In addition, incremental filling ensures a high degree of conversion without jeopardizing the mechanical properties of the composite resin as light-curing irradiance diminishes with increasing resin thickness^5,6^.

Bulk-fill composite resins have been introduced to simplify dental procedures and save chair time compared with incremental techniques that use conventional composite resins^7^. According to the manufacturers of bulk-fill resins, bulk-fill composites have modified monomers that help reduce polymerization shrinkage stress, while a lower filler amount or greater filler size facilitates light transmission due to reduction of light scattering at the filler-matrix interface^8,9^. These modified properties of bulk-fill composites ensure fillings 4–5 mm thick in bulk. Nevertheless, any type of composite resin can entrap voids, creating gaps between the resin and the tooth structure during placement^7^. Voids can be more readily entrapped when a large volume of resin is packed in a confined cavity. In addition, microleakage between the tooth and resin is associated with reduced mechanical strength, bond strength, discoloration and secondary caries^10,11^.

Hand-held vibratory devices have been developed to facilitate composite resin adaptation and handling. Vibration reportedly reduces the viscosity of the resin, allowing intimate adaptation of the composite to the cavity^12^; packable materials with greater viscosity can be used in a manner similar to that of flowable resin, without the drawbacks of high polymerization shrinkage and poor mechanical properties^13^. Most studies of the application of vibration during restoration have used the SonicFill system (Kerr Corp., Orange, CA, USA), which is a sonically activated handpiece that delivers a packable composite resin with a low viscosity. However, it is difficult to apply these results to different bulk-fill resins because the SonicFill system uses a special type of compule to ensure compatibility. Another option for applying vibration in composite resin restoration is the use of a vibratory resin applicator, but little relevant research has been reported. No studies have evaluated the adaptation of composite resin to the cavity during incremental or bulk filling with or without vibration. The aim of the present study was to evaluate the influence of vibration (no vibration vs. vibration) and resin-filling techniques at different resin thicknesses (incremental filling vs. bulk filling) on cavity adaptation by measuring void formation at various sites within the cavity. The null hypotheses were that void formation would not differ between conventional placement without vibration and modified placement with vibration, and that neither incremental filling nor bulk filling would affect void formation.

## Method and materials

### Rheological measurement of composite resin

A nano-hybrid bulk-fill composite resin (Filtek Bulk Fill [FB] Posterior Restorative, lot No. 4864A3; 3M ESPE, St. Paul, MN, USA) was used in this study as restorative material. The inorganic filler loading of the composite resin accounted for 76.5% of the resin by weight and 58.4% by volume. A dynamic frequency sweep test was performed to determine the viscoelastic change of the composite resin under oscillating shear using a rotational rheometer (ARES-G2; TA instrument, New Castle, DE, USA) equipped with 8 mm parallel plates set to a distance of 2 mm. The storage modulus G′, loss modulus G″, and complex viscosity η^*^ were measured at 30℃ and at frequencies from 0.1 to 100 Hz. The measurements were performed at a shear strain of 2%, which is close to the limit of linear viscoelastic resin and similar to the amplitude of vibration device and specimen thickness (0.05 mm and 2 mm)^14^.

### Vibration measurement with a scanning laser Doppler vibrometer

**Figure 1:**
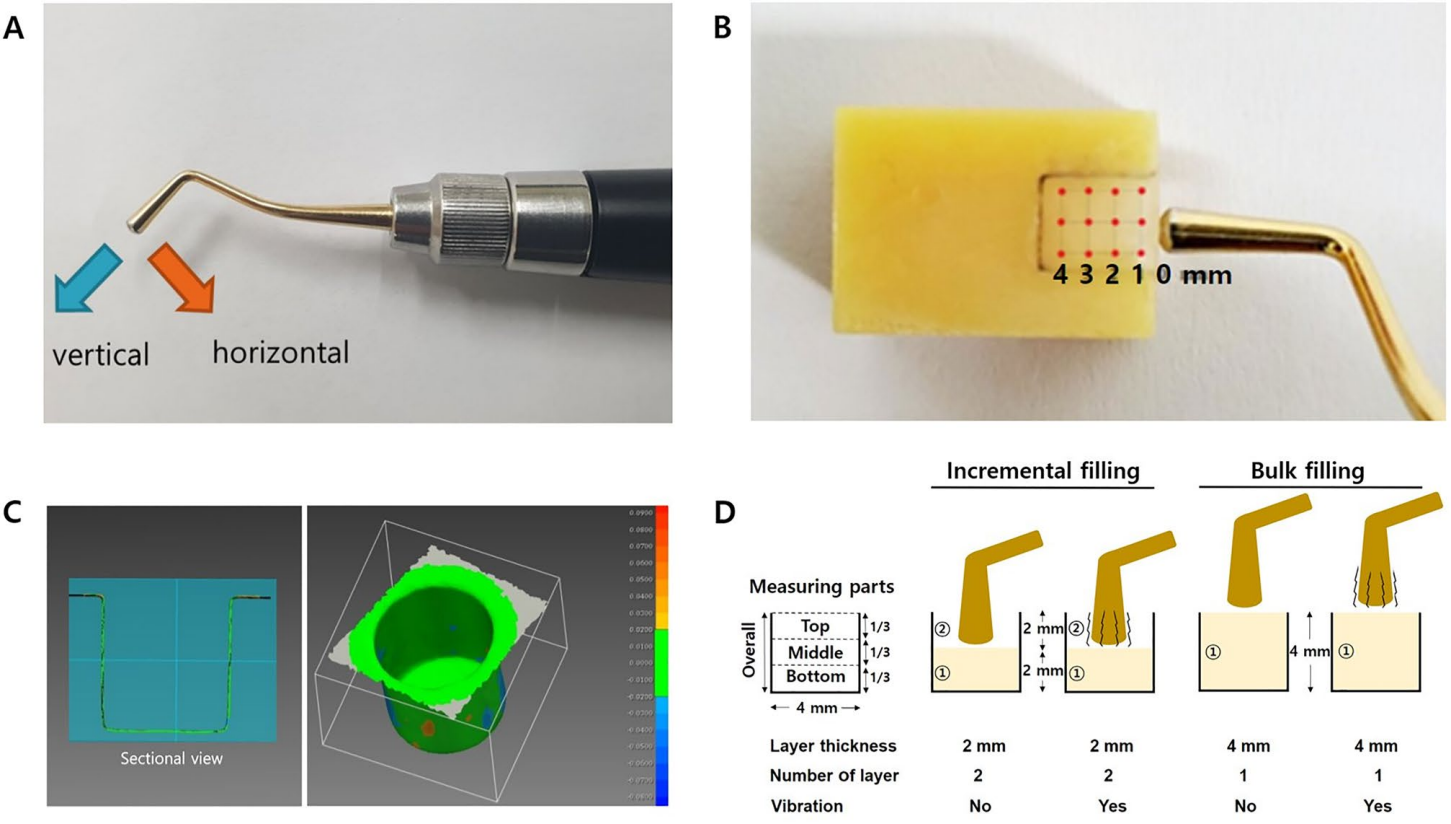
Experimental set-up. (**A**) Vibratory resin applicator with a rounded-end tip. The vibration frequency of device was measured vertically and horizontally to the tip. (**B**) Measurement points 1-mm apart placed on the resin surface using SLDV software^16^. (**C**) Representative deviation colour map of milled cylindrical cavities. Green indicates a value equal to or less than a deviation of ± 20 µm. (**D**) Study group for evaluation of composite resin adaptation to the cavity.

A vibration-generating resin applicator (COMO; B&L Biotech, Ansan, Korea) was used to transmit vibration energy on composite resin. The device is a battery-operated motor-driven vibrator specifically designed for manipulation of dental resin. A rounded-end tip 2 mm in diameter was used among various changeable tips. A scanning laser Doppler vibrometer (SLDV) (Optomet GmbH, Darmstadt, Germany) was used to measure the vibration of the device. The SLDV uses a short-wavelength infrared laser, and the Doppler shift of the reflected laser beam is used to measure vibration velocity^15^. After fixing the device in place with a rounded-end tip (2 mm diameter), the SLDV beam was focused on the vibrating tip to measure the frequency and amplitude of oscillation, both perpendicularly and horizontally (Fig. 1A). Each scan lasted approximately 10 s, with an interval of 20 s between scans. Five repeat vibrometer scans were performed.

### Resin vibration measurement using a scanning laser Doppler vibrometer

A block with a cavity (5 mm wide, 4 mm high, and 4 mm deep) was fabricated using a 3D printer (IMC; Carima, Seoul, Korea). Two sides of the cavity were opened to allow access for the SLDV beam and the vibration device. A mesh of 12 measurement points, arranged in a grid with 1 mm between points, from the open surface was placed on the resin-filled surface using the SLDV (Fig. 1B). The vibrator tip was provided with vibration energy on the surface of the open side of the filled resin. The vibration of the entire grid on the composite resin surface was scanned and the vibration velocities were recorded (n = 5). The averaged values of the three measuring points at each depth were compared to understand the general trend of vibration effect according to resin depth. Dedicated SLDV software^16^ (OptoSCAN; Optomet GmbH) displayed the vibration pattern in a colour map.

### Adaptation evaluation using micro-CT

#### Specimen preparation

Standardized cylindrical class 1 cavities (4 mm diameter, 4 mm depth) were milled on resin-based hybrid ceramic CAD/CAM block (Mazic Duro; Vericom, Anyang, Korea) using a computerized numerical control milling machine (A-PRO MILL; Namsun, Daejeon, Korea). The milled cylindrical cavities were digitized using a 3D scanner (T500; Medit, Seoul, Korea) and saved as STL files. The STL data of the cavities were superimposed on each other using metrology software^17^ (PointShape Inspector 2.16; DREAMTNS, Seongnam, Korea). The deviation of each cavity was within ± 20 µm, verifying the dimensional accuracy of the milled cavities (Fig. 1C).

#### Restorative procedure

The cavities were roughened with 50 µm aluminium oxide (blasting medium; Dentaurum GmbH & Co. KG, Ispringen, Germany), cleaned with copious water and air dried. The cavities were then pretreated with a silane coupling agent (Porcelain Primer; Bisco, Schaumburg, IL, USA) for 60 s and air dried. A bonding agent (Single Bond Universal; 3M ESPE) was applied to each cavity and light-cured for 10 s (Elipar DeepCure-S; 3M ESPE) after adequate air drying until no visible movement of the bonding agent was observed. Filtek Bulk Fill (3M ESPE) was placed into the cavities using a delivery gun. The tip of the resin compule was initially placed in contact with the cavity floor, and the resin was delivered slowly while the gun was moved away from the cavity floor to minimize entrapment of air in the resin. Resin filling was performed with two different layering techniques (incremental filling with two 2-mm-thick layers and bulk-filling with a single 4-mm-thick layer). Each layer was packed for 10 s with two strokes per second using a 2-mm-diameter tip on the vibratory device specifically designed for resin application (COMO; B&L Biotech) with or without vibration (no vibration vs. vibration) (n = 10) (Fig. 1D). Light-curing was performed for 20 s after placement of each layer.

#### Evaluation of surface adaptation and void volume using micro-CT

Each sample was scanned using high-resolution micro-CT (Skyscan 1273; Bruker, Kontich, Belgium). Exposure parameters were set at a tube voltage of 120 kVp, a current of 125 μA, a voxel size of 9.88 μm, a 0.4° rotation step, and an average of three frames with an exposure time of 42 min. Aluminium and copper filters were used to suppress beam-hardening artifacts. The 2D projection images were transformed into 3D volumes using a reconstruction program^18^ (NRecon, ver. 1.7.5.1; Bruker).

A two-dimensional CT image of the cavity floor was selected and the percentage of the entire floor surface area occupied by void surface area was calculated to evaluate the surface adaptation at the cavity floor using analysis software^19^ (CTAn, ver. 1.18.9.0; Bruker).

The total void volume (void volume per total cavity volume, %) and void volume of three separate parts (bottom, middle, and top) were evaluated using CTAn. The bottom part of the specimen was defined as the cavity floor to a height of 1.3 mm, the middle part as 1.3–2.6 mm, and the top part as 2.6–3.9 mm (Fig. 1D). The void volume of each part and the total void volume were compared according to vibration application and filling technique. The 3D void distribution was visualized using rendering software^20^ (CTVox, ver. 3.3.0; Bruker).

#### Statistical analysis

Levene’s test was used to assess the equality of variances and the Shapiro–Wilk test was used to verify the normality of each variable. The median values of the resin vibration measured by the SLDV and the median values of the 2D void surface and 3D void volume (%) were analysed using the Kruskal–Wallis test, followed by a post hoc Mann–Whitney U test with Bonferroni correction for pairwise comparisons. The void volume between filling techniques was compared using Mann–Whitney U tests. All tests were conducted at a level of significance of 0.05. Statistical analyses were performed using IBM SPSS Statistics, v25 (IBM Corp., Armonk, NY, USA).

## Results

### Rheological measurements

**Figure 2:**
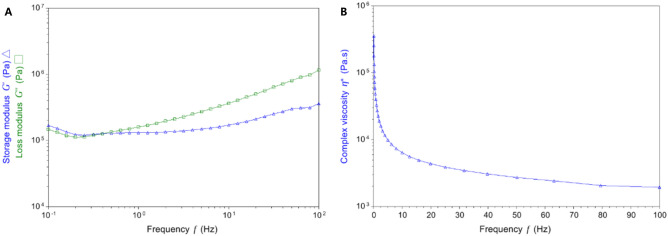
(**A**) Storage modulus G′, loss modulus G″, and (**B**) complex viscosity η^*^ as a function of frequency.

Both the storage modulus G′ and loss modulus G″ increased with frequency. The Filtek Bulk Fill resin showed pseudoplasticity; the complex viscosity η^*^ of the composite resin decreased with increasing frequency, from 32,918 Pa·s at 1 Hz to 1936 Pa·s at 100 Hz (Table 1; Fig. 2).

**Table 1:** Changes in storage modulus, loss modulus, and complex viscosity as a function of frequency.

Frequency (Hz)	Storage modulus (Pa)	Loss modulus (Pa)	Complex viscosity (Pa·s)
0.1	168,920	147,987	357,423
1	131,278	159,829	32,918
10	170,599	365,105	6,414
100	361,987	1,161,610	1936

### Evaluation of device frequency and amplitude

**Figure 3:**
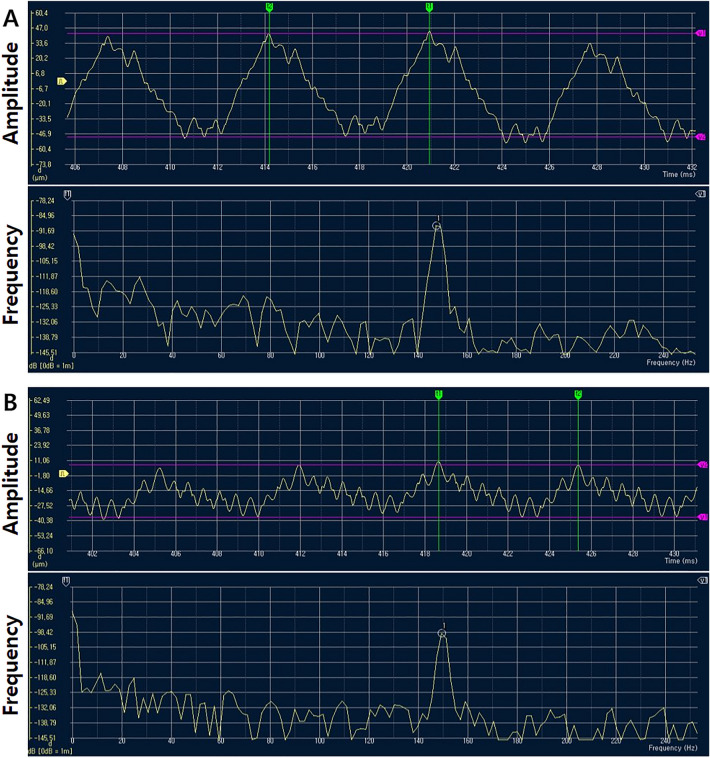
Frequency and amplitude of the vibratory resin applicator. (**A**) Vertical direction. (**B**) Horizontal direction.

The vertical and horizontal frequencies of COMO were 149 Hz, while the vertical and horizontal amplitudes of COMO were 50.5 µm and 26.4 µm (Fig. 3).

### Evaluation of resin vibration using SLDV

**Figure 4:**
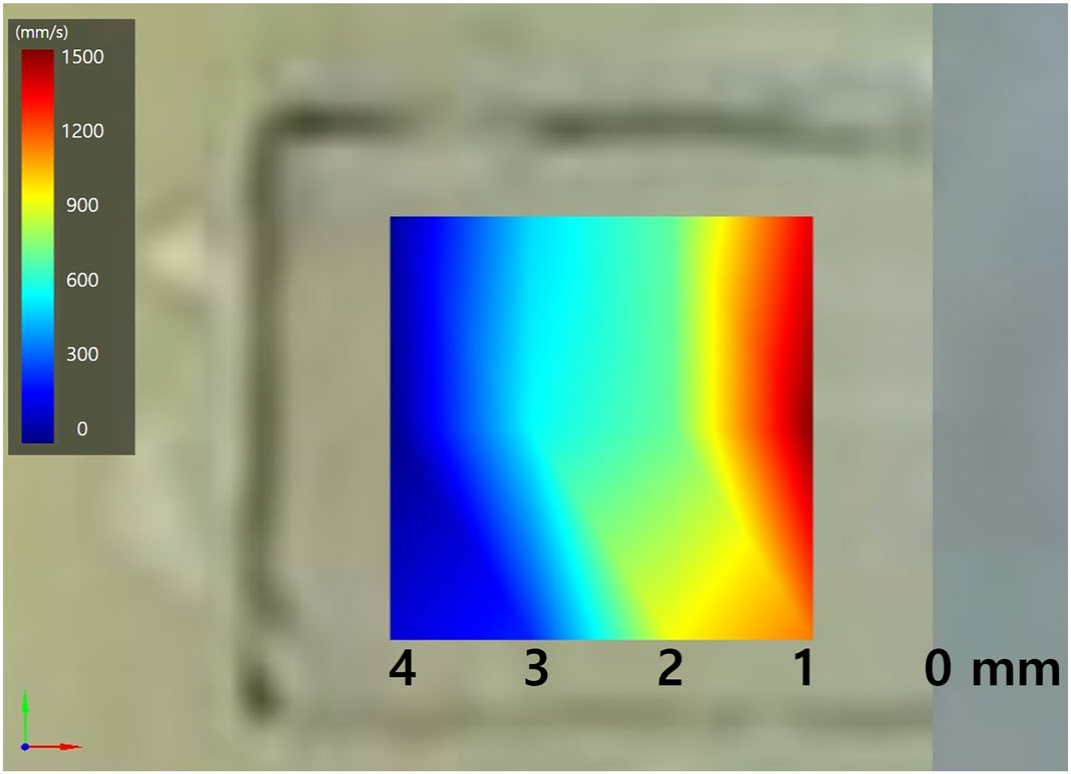
Representative colour map of composite resin vibration.

The velocity of vibration decreased as the depth of the resin increased. The vibration effect, as indicated by velocity of vibration, was significantly reduced as the depth of the resin increased (*p* < 0.001) (Table 2). Figure 4 provides a representative colour map of composite resin vibrations scanned by SLDV. Vibration energy was able to propagate to a resin depth of approximately 2 mm, and little vibration remained at 4 mm.

**Table 2:** Resin vibration at various distances from the resin applicator tip.

Distance from tip	1 mm	2 mm	3 mm	4 mm	χ^2^	*p*
Vibration velocity (mm/s)	1804 A (1325–1937)	974 B (766–313)	462 C (322–769)	68 D (43–172)	48.75	< 0.001

Interquartile ranges (first quartile, third quartile) are in parentheses.

Different uppercase letters indicate statistically significant difference (row) in the Kruskal–Wallis and multiple comparisons by Mann–Whitney U test, *p* < 0.05).

*χ*^*2*^, chi-square; *p*, p-value.

### Micro-CT analysis

#### Evaluation of surface adaptation at the cavity floor

**Figure 5:**
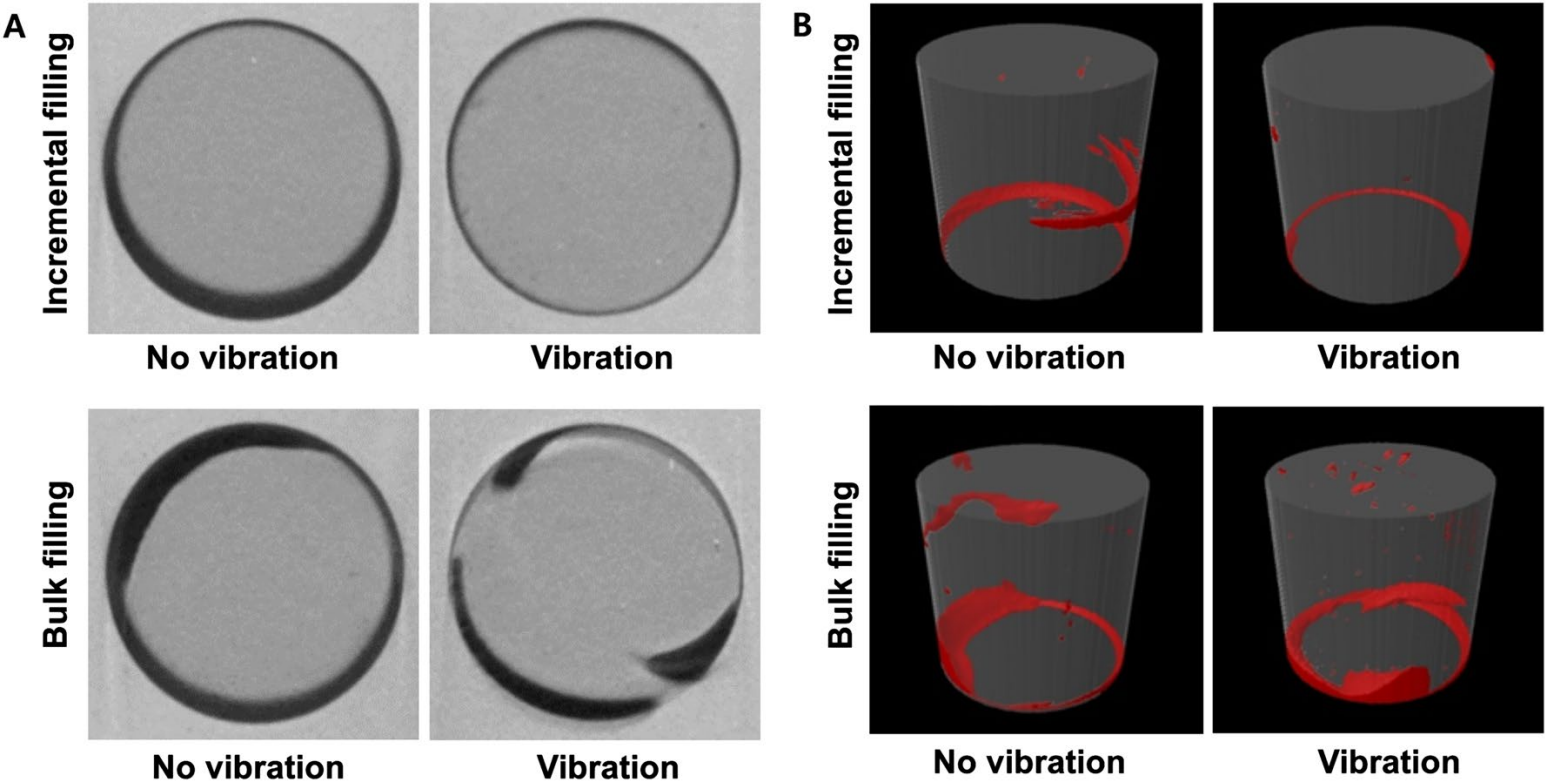
Representative sample of (**A**) a 2D micro-CT image at the bottom surface of the cavity and (**B**) a 3D rendering image of each group.

Table 3 presents the medians and interquartile ranges of surface adaptation (surface area % at the pulpal floor). Vibration was associated with a significantly smaller void surface area in the incremental filling (*p* = 0.035), while no difference was observed in the bulk filling (*p* = 0.631). The differences between incremental filling and bulk filling were not statistically significant with vibration (*p* = 0.131) and without vibration (*p* = 0.762). Two-dimensional micro-CT images revealed that voids formed most frequently around the corner of the cavity floor, with a marked decrease of void tendency in incremental filling with vibration (Fig. 5A).

**Table 3:** Surface adaptation (void area %) at the cavity floor.

	No vibration	Vibration	*U*	*p*
Incremental filling	15.38 (14.03–21.17)	8.72* (5.30–15.47)	22	0.035
Bulk filling	17.15 (11.63–23.05)	15.49 (12.43–20.84)	43	0.631
*U*	46	30		
*p*	0.762	0.131		

Interquartile ranges (first quartile, third quartile) are in parentheses.

An asterisk indicates significantly higher void volumes within the same filling technique between no vibration and vibration (row) (Mann–Whitney U test, *p* < 0.05).

*U,* U-value; *p*, p-value.

#### Evaluation of 3D void volume

**Figure 6:**
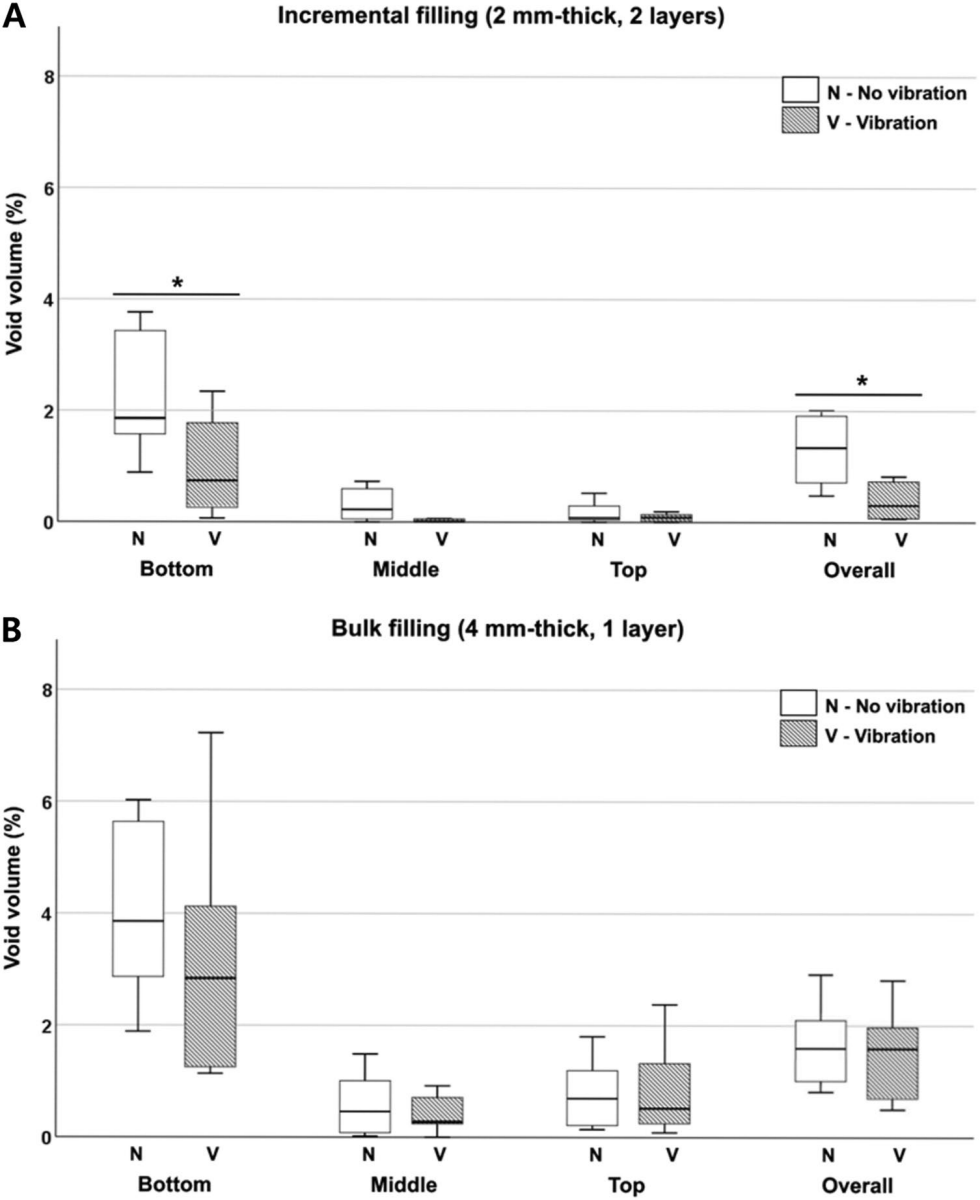
Void volume (%) in (**A**) incremental fillings and (**B**) bulk fillings with or without vibration in different cavity parts.

Images rendered in 3D showed smaller overall and internal voids at the bottom surface corner in incremental fillings compared with bulk fillings, particularly when vibration was applied. Some voids were found at the middle of the cavity in some of the incremental fillings (Fig. 5B). A significantly higher void volume (%) was observed in the bottom part both in the incremental and bulk filling groups, regardless of vibration mode (*p* < 0.001), while no significant differences were found between the middle and top parts (*p* > 0.05) (Table 4; Fig. 6). When compared with conventional applications, the vibration mode significantly reduced void volume in the bottom part of the incremental filling (*p* = 0.023) but not in the bottom part of the bulk filling (*p* = 0.226). The overall void volume was significantly lower only in the incremental filling when the composite was filled using the vibration mode (*p* = 0.003). Regardless of vibration application, compared with a bulk filling, the void volume in the bottom part was significantly reduced in the incremental filling (*p* = 0.028 for no vibration and *p* = 0.005 for vibration) (Table 5).

**Table 4:** Void volume (%) of incremental fillings and bulk fillings in different cavity parts between no vibration and vibration.

Cavity part	No vibration	Vibration	*U*	*p*
**Incremental filling**				
Bottom	1.85 A (1.57–3.49)	0.74 A* (0.23–1.91)	20	0.023
Middle	0.23 B (0.04–0.63)	0.01 B (0–0.06)	26	0.070
Top	0.08 B (0.03–0.35)	0.09 B (0.02–0.15)	43	0.597
χ^2^	18.91	15.69		
*p*	< 0.001	< 0.001		
Overall	1.34 (0.69–1.94)	0.31* (0.08–0.75)	11	0.003
**Bulk filling**				
Bottom	3.86 A (2.15–5.67)	2.84 A (1.25–4.36)	34	0.226
Middle	0.46 B (0.08–1.01)	0.28 B (0.18–0.76)	47	0.821
Top	0.70 B (0.21–1.20)	0.52 B (0.24–1.49)	54	0.762
χ^2^	17.61	15.73		
*p*	< 0.001	< 0.001		
Overall	1.62 (1.08–2.23)	1.59 (0.67–2.07)	40	0.450

Interquartile ranges (first quartile, third quartile) are in parentheses.

Different uppercase letters indicate statistically significant difference between bottom, middle, and top parts (column) (Kruskal–Wallis and multiple comparisons by Mann–Whitney U tests, *p* < 0.05).

An asterisk indicates significantly lower void volume within same part between no vibration and vibration (row) (Mann–Whitney U tests, *p* < 0.05).

*χ*^*2*^, chi-square; *U,* U-value; *p,* p-value.

**Table 5:** Void volume (%) in the bottom, middle, top, and overall cavity for incremental and bulk fillings.

Cavity part	No vibration	Vibration
**Bottom**		
Incremental filling	1.85* (1.57–3.49)	0.74* (0.23–1.91)
Bulk filling	3.86 (2.71–5.67)	2.84 (1.25–4.36)
*U*	80	87
*p*	0.028	0.005
**Middle**		
Incremental filling	0.23 (0.04–0.63)	0.01* (0–0.06)
Bulk filling	0.46 (0.08–1.01)	0.28 (0.18–0.76)
*U*	59	78
*p*	0.496	0.034
**Top**		
Incremental filling	0.08* (0.03–0.35)	0.09* (0.02–0.15)
Bulk filling	0.70 (0.21–1.2)	0.52 (0.24–1.49)
*U*	85	92
*p*	0.008	0.001
**Overall**		
Incremental filling	1.34 (0.69–1.94)	0.31* (0.08–0.75)
Bulk filling	1.62 (1.08–2.23)	1.59 (0.67–2.07)
*U*	63	86
*p*	0.353	0.007

Interquartile ranges (first quartile, third quartile) are in parentheses.

An asterisk indicates significantly lower void volume within same part between incremental fillings and bulk fillings (column) (Mann–Whitney U test, *p* < 0.05).

*U,* U-value; *p,* p-value.

## Discussion

Adaptation of bulk-fill composite resin was analysed by evaluating the void area at the cavity floor and the void volume in the bottom, middle, and top-third of the cavity using a non-destructive technique and micro-CT. The findings of the present study showed that void formation varied depending on layering methods at different resin thicknesses (incremental filling vs. bulk filling) and vibration application (no vibration vs. vibration), rejecting the null hypotheses.

Regardless of vibration application and resin thickness, voids were more frequently observed in the bottom part of the cavities compared with the middle and top parts. These findings were in agreement with a previous study^14^, confirming that the bottom part is most likely to be free of voids. In the present study, vibration resulted in significantly improved surface adaptation at the cavity floor and reduced void volumes, particularly in the bottom part, when composite resin was placed in two 2-mm-thick layers through incremental filling. A correlation between gap formation at the pulpal floor and post-operative sensitivity has long been established^21^; using a vibration device combined with incremental layering might help alleviate post-operative sensitivity. As the presence of an internal void within the composite resin reduced the durability and weakened the mechanical properties, possibly leading to fractures and failure of resin restoration^22^, the use of a vibration device in composite resin placement appears to be useful for increasing the strength and durability of resin restoration. Han et al. also studied the effect of the device used in the present study on resin adaptation^14^. The authors reported a tendency of FB to result in fewer gaps forming with vibration, although the difference was not statistically significant. The difference between the present and previous findings could be explained by variability in experimental features, such as number of samples, cavity geometry, design, and resin type (syringe vs. compule type).

Within the bottom part of the cavity, voids were more readily detected at the cavity floor, particularly around the line angle at the junction of the axial wall and cavity floor. This might be associated with cavity geometry; the cavity had a sharp line angle that represents the most challenging situation for intimate adaptation of composite resin to the line angle. In addition to superior distribution of shrinkage stress, rounded internal angles would be also favourable for resin adaptation. Flowable composite resins might allow for superior adaptation to cavity walls due to their lower viscosity; applying flowable resin as a liner should therefore enhance adaptation and reduce microleakage^23^. Use of a flowable resin as a liner is also effective in reducing cusp deflection due to its low elastic modulus^24^. One other method of reducing the viscosity of composite resin is preheating before delivery^25^. As with vibration, preheating improves handling and increases adaptation, with none of the mechanical disadvantages. Although multiple studies have reported superior adaptation and decreased gap formation with preheated resin composite compared with resin at room temperature^26–28^, injecting heated resin into the cavity might not favour pulpal health^29^.

An SLDV is a non-invasive device for measuring the instantaneous velocity of vibrating objects using the Doppler shift of laser light^15^. SLDVs have replaced accelerometers and other surface-contacting sensors in the measurement of vibrating objects due to the non-contacting nature of the instrument. They have been used to measure the vibration patterns of dental devices, including an ultrasonic scaler^30^, high-speed handpiece^31^, endosonic file^32^, and powered tooth brush^33^. In the present study, the frequency and vertical and horizontal amplitudes of the COMO vibratory resin applicator measured by SLDV were approximately 149 Hz and 50.5 µm and 26.4 µm, respectively. For rheology tests, the complex viscosity of the bulk fill decreased significantly as the vibration frequency increased. This phenomenon is known as pseudoplasticity and is a common characteristic of composite resin caused by molecular repositioning and separation under shear stress^34^. As the frequency exceeded 100 Hz, complex viscosity converged to approximately 2000 Pa·s, which means that the degree of viscosity reduction due to the vibrator might be similar or slightly lower if the vibration frequency is greater than 100 Hz. Although vibration effectively reduced void formation in the bottom part of the incremental filling, its effect was negligible in bulk filling. The 4-mm-thick resin in the bulk filling was too thick for the vibration energy to be delivered effectively to the resin, as evidenced by the colour map obtained by the SLDV, which showed a gradual reduction of the vibration effect as the distance from the applicator tip increased. The limited vibration effect beyond a depth of 2 mm accounts for the contrasting findings between incremental and bulk filling.

Previous studies of the use of vibration devices for composite resin restoration report conflicting outcomes. Most studies of the effect of vibration on resin restoration employed the SonicFill system. According to the manufacturer, the increased flowability due to vibration is intended to achieve more precise adaptation to cavity walls, but these results are controversial. One study that evaluated microleakage in class II restoration using dye penetration reported that the SonicFill had the lowest microleakage values among tested groups^35^. In contrast, studies of gap formation and microleakage reported that the SonicFill system produced no specific differences compared with other bulk-fill resins^7,36^ or conventional resins with incremental layering^37^. A study evaluating the internal adaptation and gap formation using several bulk-fill resins and a conventional resin as a control found that the SonicFill system produced significantly larger gaps and less adaptation to cavities compared with other tested resins^38^. Other studies reported that the sonic insertion method has no positive effect on void reduction during delivery of resin composites^12,26^. Given that the SonicFill is a sonically activated resin-delivery system, no additional vibration can be applied to condensed resin after it is placed in the cavity. In contrast, the device used in this study was designed to provide vibration energy with a packing motion after the resin is placed in the cavity. Because the mode of vibration differs between the two devices, it is not appropriate to directly compare the results of the present study to those of studies using SonicFill.

The void volumes of all areas of interests were significantly greater in 4-mm bulk filling compared with those of 2-mm incremental filling, irrespective of vibration application. Although a greater reduction was seen with vibration, incremental filling of the bulk-fill resin using a conventional method also effectively reduced void formation. This can be explained by easier adaptation and relatively greater chances of void escape during placement of a relatively smaller amount of composite resin. In addition, a smaller amount of composite resin in each layer is associated with less shrinkage stress and exothermic heat generation during polymerization^39^. The placement of bulk-fill composite resins in incremental layers could be recommended over bulk filling to minimize void formation and possible thermal damage to the pulp while still ensuring a reliable interface between teeth and resin restoration. Some voids were found at the middle of the cavity in the incremental filling group, but these were assumed to be entrapped voids between the incremental layers. Meticulous placement of resin increments should eliminate potential discrepancy between layers.

The findings of this study should be interpreted with caution given certain limitations. Only a nano-hybrid resin, FB, was used, and the effect of vibration on different composite resins of various viscosity should be subjected to further study because the effect of vibration would not be identical in other composite resin with varying compositions. Standardized cylindrical class I cavities were prepared for restoration, but different cavity designs can affect vibration propagation and composite resin adaptation. We were unable to directly compare the effects of vibratory device–related variables, such as vibration frequency, amplitude, and applicator tip design (all of which can alter vibrational properties), because no other vibratory devices designed for resin placement were available. However, our findings suggest a potential application of vibration during composite resin placement in terms of reducing void formation at the interface between the cavity and resin and within composite resin. Further studies are needed to validate the effect of vibration in composite resin restoration.

## Conclusion

Within the limitations of this study, the following conclusions can be made.

- Void formation varied depending on cavity area, layer thickness, and vibration application.
- Void volume was markedly higher in the bottom part of the cavity, particularly around the corner of the cavity floor, in both layer thicknesses compared with the middle and top parts, regardless of vibration application.
- When vibration was not applied, incremental filling with two 2-mm-thick layers resulted in a smaller void volume in the bottom third of the cavity than did bulk filling with a single 4-mm-thick layer.
- Vibration further reduced the amount of void formation in the bottom third of the cavity during incremental filling, but no effect was seen during bulk filling.

## Data Availability

The datasets generated during the current study are available from the corresponding author on reasonable request.
